# Pregnancy resolutions among pregnant teens: termination, parenting or adoption?

**DOI:** 10.1186/s12884-014-0421-z

**Published:** 2014-12-19

**Authors:** Alice Yuen Loke, Pui-ling Lam

**Affiliations:** Professor, School of Nursing, The Hong Kong Polytechnic University, Hong Kong, China; Registered Nurse, Maternal & Child Health Centre, Department of Health, Hong Kong, China

**Keywords:** Teenage pregnancy, Pregnancy resolution, Decision making, Adolescent nursing

## Abstract

**Background:**

Teenagers are unprepared to face or to deal with an unexpected pregnancy. Adolescents do not necessarily possess the cognitive ability needed to clearly evaluate such a situation or to determine how to resolve their pregnancy. This study seeks to shed light on what pregnant adolescents consider when coming to a decision about what to do about their pregnancy.

**Methods:**

In-depth interviews were conducted among a purposive sample of Hong Kong Chinese women recruited from a Maternal and Child Health Centre, who had a history of being pregnant in their teens and out of wedlock. Interviews were conducted to explore the considerations surrounding their decision on how to resolve their pregnancy.

**Results:**

A total of nine women were interviewed. An analysis of the interview transcripts revealed that to arrive at a decision on what to do about their pregnancy, pregnant teens took into consideration their relationship with their boyfriend, their family’s advice or support, practical considerations, their personal values in life, and views on adoption.

**Conclusions:**

The results of this study results highlighted that during this life-altering event for adolescents, an open discussion should take place among all of the parties concerned. A better understanding of each party’s perspective would allow for better decision making on the resolution of the pregnancy. Health professionals or social workers are there to help pregnant adolescents, romantic partners, and family members make informed choices on how to resolve the pregnancy.

## Background

The rate at which adolescents bear children is reported to be low in Hong Kong, at 3 per 1,000 [1]. However, this rate may be misleading, since in Hong Kong a pregnancy can be terminated only if two physicians both agree that the pregnancy would pose a risk to the life of the pregnant woman, the fetus is at a high risk of suffering from a serious deformity or disabilities, the woman in question is 16 years old or younger, or the woman was the victim of rape, incest, or abuse. However, the social stigma of being an unmarried woman with a child is still high, and children born outside of a marriage are often regarded as illegitimate and face discrimination in HongKong. Such social pressure, coupled with a law that stipulates that performing an abortion oneself makes one liable to imprisonment, has caused many unmarried women to cross the border to mainland China to terminate their pregnancy. There are no official statistics for these rates.

Teenagers are unprepared to face or deal with their own unexpected pregnancy or that of their girlfriend. A study found that many of the adolescents who had dating experience (66.7%) revealed that they would not know what to do in case of pregnancy, and that the majority of the sexually active adolescents (81.8%) admitted to having worried about a possible pregnancy [2].

When facing an unexpected pregnancy, teenage girls experience fear, confusion, guilt and worry [3,4]. Some may even deny the existence of their pregnancy [5]. The fear of being stigmatized and revealing the pregnancy to other people may prevent them from receiving early advice [4,5]. Their parents and boyfriends may put pressure on the pregnancy and contribute to their resolution decision [3,4].

Adolescents may not necessarily possess the cognitive ability needed to clearly evaluate their situations and to make rational decisions [6]. On discovering that they are pregnant, some make the immediate decision to terminate the pregnancy without considering other options [3,5]. Among those who carry their pregnancy to term, some choose to terminate parental rights and place the newborn for adoption, while others choose parenting.

When facing an unintended pregnancy, the romantic partner is typically the first person approached by a pregnant teenager in the process of making the decision. Pregnant teenagers may also turn to their mother for advice [7]. The attitudes, relationships, and support of these people greatly influence a pregnant teen’s decision on whether to undergo an abortion or become a parent [8,9].

The personal values and attitudes of pregnant adolescents also have a bearing on the decision that they make. Adolescents who choose parenting commonly have negative views of abortion or adoption. Those who believe that adoption would result in severe psychological distress tend to refuse to give the baby up for adoption. Some believe that keeping the baby means taking responsibility for their mistake [5]. Adolescents who value “family and kinship” have fewer practical concerns about the costs and difficulties of parenting. Those who choose abortion have less concern for “life” and “family and kinship” [3].

A few studies have been conducted in the West to explore the decision-making process that teenagers go through when trying to come to a decision about their pregnancy [10-12]. A study was conducted to explore the lived experiences of young Chinese women during their unintended pregnancy [13], and another study focused on explaining the decision made by young Chinese women to keep their baby and to take up the responsibilities of being a single mother [14]. However, there has been no study on the different decisions that young Chinese women make concerning their pregnancy. It is unclear how teenage girls, particularly Chinese adolescents in Hong Kong, arrive at their pregnancy resolution.

## Methods

### Study design

This study aims to explore the considerations surrounding the pregnancy resolution of women who were pregnant in their teens while single. An understanding about pregnancy resolution from the perspectives of pregnant adolescents will enable sensible advice and support to be delivered to pregnant adolescents and their families. With adolescents having unique life values and considerations that might influence their pregnancy resolution, a qualitative research study was adopted.

#### Target sample and setting

It was the original intention of this study to recruit pregnant teens for interviews. However, recruiting informants proved to be very difficult. Organizations that provide services for pregnant adolescents were approached; however, as gatekeepers to protect the interests of their vulnerable clients, all of these organizations denied us access.

It was then decided to recruit informants who had experienced an unexpected pregnancy within the last two years when they were aged 19 or younger. A purposive sample was recruited from a Maternal and Child Health Centre (MCHC) of the Department of Health. MCHCs provide antenatal and postnatal care services to women, as well as sexual and reproductive health, family planning, and cervical cancer screening services. Midwives and nurses work in these clinics, with the midwives usually being the in-charge of the clinic.

### Data collection

#### Interview guide

An interview guide that included the issues to be explored was drawn up in advance. These issues included the possible considerations related to family relationships and the influence of significant others on the pregnancy resolution. The interview guide was approved by three experts, including a social worker with extensive experience working with pregnant teenagers, a nurse specializing in maternal care, and a university professor focusing on women’s health. Two questions on “child care and financial considerations” and “personal views on adoption” were added to the interview guide according to the experts’ suggestions.

Pilot interviews with two informants contacted through friends of one of the researchers were conducted. The pilot study allowed the researcher to test the interview guide and process. The transcriptions and the preliminary coding of the data were examined by the two researchers.

### Interviews

Face-to-face semi-structured interviews were conducted. The interviews were conducted by the research-interviewer who is not an employee of the clinic. The interviews were conducted in Cantonese and took place in an interview room at the MCHC. The room was a small private room, located away from the waiting hall, and a light indicating “interview in progress” was turned on to avoid being disturbed. This was done to provide comfort, privacy, and a quiet environment for conversation and clear audio-recording of the interviews.

#### Ethical considerations

Before conducting the study, ethical approval was obtained from the Ethical Review and Approval Committee, The Hong Kong Polytechnic University. Information related to the study was given to each informant. Those who were willing to take part in the study were interviewed immediately after their written consent to do so was obtained, or were scheduled for an interview according to the preference of the informants, usually within two weeks. As the audio recordings, notes, or interview transcripts contained sensitive and personal information about the informants, a pseudonym was assigned to each informant. All of the information was in a locked filing cabinet to assured confidentiality.

As the informants might recall the stressful experience of teenage pregnancy, measures were taken to safeguard their emotional well-being during the interview. The informants were informed about the voluntary nature of the research, that their decision on whether or not they agreed to be interviewed would not affect the treatment that they received from the clinic, and about the possibility of withdrawal at any stage, especially when feeling emotional discomfort. Support would be given during the interviews, and interviewees would be referred to counselling services if they needed further emotional support. None of the interviewees required emotional support after the interviews.

#### Data analysis

The informants’ demographic data were collected using a demographic sheet. The interviews were audio-recorded to “eliminate errors of memory”. The interviews were transcribed within two weeks to obtain the most comprehensive and accurate description. In order to maintain dependability, the researcher-interviewer repeatedly listened to the tape during transcription and proofread the verbatim transcription for accuracy. The transcriptions were typed in Chinese.

Conventional content analysis of qualitative data was used to handle the data [15,16]. The researchers read the data repeatedly to obtain a sense of the whole; the data were read word-by-word and the text was highlighted. Codes were then derived, existing codes were revised if new codes emerged (further coding and recoding), and further analysis was conducted to develop themes that could authentically and accurately represent the topic being studied [16].

Throughout the whole data analysis process, in order to maintain accuracy the language that was used in the transcription of data, with the highlighting of text, codes, and emergent themes, the language that was used was Chinese, the language with which the researcher are most familiar. It is only at the stage of writing up the manuscript that the themes were translated into English.

#### Rigor

The two researchers independently read through the interview transcripts of each informant several times so as to make sense of the data and obtain a sense of the whole. Significant statements pertaining to the situation, choices, and decision of the women were extracted and defined as phrases. The primary patterns were organized into themes, which were then compared with the original description. The two sets of analyzed themes were compared and discussed until a consensus was reached, and the themes were combined, summarized and classified accordingly. Study rigor was enhanced through a member check of the emerging clusters with the informants.

### Study Results

#### Characteristics of the informants

In the months from August to October 2009, nurses who worked in the MCHC helped to review the records of current clients to identify and recruit those eligible as informants. A total of twelve women were considered eligible and invited for interviews. However, one repeatedly missed the appointments, one lost contact, and one was advised by her mother to refuse to be interviewed. A total of nine informants were successfully interviewed. The interviews lasted for 25 to 45 minutes.

All nine informants were Chinese women born in Hong Kong who had experienced an unintended first pregnancy when they were single in their teens. Seven informants were 17 years old when they first became pregnant, one was 18, and another 15. Four of them had been studying in secondary school, and one had been working as clerk, and one as a waitress. Three were not engaged in either school or work at the time of the pregnancy. All of the four who were in school discontinued their schooling after their pregnancy. Four were raised in single-parent families. GiGi, Helen, and Lucy moved out of their family home at the time of the pregnancy. Both GiGi and Lucy came from divorced families and described their families as uncaring.

Among the nine informants, four changed their initial choice of what to do about their pregnancy during the course of the pregnancy. Five informants finally chose to undergo an abortion (Candy, Helen, Ivy, Jenny, and Kitty), and four chose either co-parenting or single parenting (Daisy, GiGi, Lucy, and Fanny). However, Fanny, who had chosen to raise the baby together with the baby’s father, was placed in a halfway house for drug addiction treatment and had to place the baby in a foster family. She eventually signed away her baby what the infant was 4 months old, when she separated from the baby’s father.

#### Considerations involved in the pregnancy resolution decision

The process of pregnancy resolution began when informants realized that they were pregnant. An analysis of the interview transcripts revealed that to arrive at a decision on how to resolve their pregnancy, several categories for consideration emerged, including the interviewee’s relationship with the boyfriend, family’s advice or support, practical considerations, personal values in life, and views on adoption.

#### Relationship with the boyfriend

All of the women had approached their boyfriend, the putative father of the baby, about their pregnancy resolution. The boyfriend was the usually the first person whom the pregnant teenager approached. The relationship with the putative father greatly influenced their pregnancy decision. Four out of the five informants who chose to undergo an abortion had a poor relationship with their boyfriend. They explained:

*He was never concerned about my feelings: instead of comforting me, he grumbled. He denied that the baby was his, and said that I had other men. We then separated. (Candy)*

*As he thought the baby was not his, and he insisted on an abortion. I was afraid that he would beat me, so I said nothing. (Helen)*

*After I told him I was pregnant, he complained, stopped talking to me, and left me a few days later. (Ivy)*

*We dated each other for only a short time, about one month; our relationship was not strong enough. (Kitty)*

The three informants who chose to co-parent were those who had a good relationship with their boyfriend (the putative father) and received support from him. They reported that their boyfriend was affectionate and supportive. They said:

*I was going to abort the baby. But we considered our relationship: since it was stable, he insisted that we keep the baby and did not want me to have an abortion. So we decided to keep the baby. (Daisy)*

*We were together and loved each other, so after discussion, we both decided to keep the baby. (Fanny)*

*I love my husband (then my boyfriend), and he was good to me. I told him that I wanted to keep the baby, and he didn’t object. (GiGi)*

Lucy was the only one who chose to become a single parent. She said that she had separated with her boyfriend before she realized that she was pregnant. When she called him to tell him the news, her boyfriend told her to get an abortion. She said:

*He said something nasty to me, and that he would stay with me just because I am pregnant. We had already separated then. He told me to go and get an abortion because he already had another girlfriend. I did not want to abort the baby, so I decided to have the baby myself. (Lucy)*

Fanny initially chose to co-parent, but finally gave the baby up for adoption when the baby was 4 months old. She said:

*I was in a drug treatment program. I surely would have taken care of the baby even though we were separated, but I had no choice: I had to put the baby up for adoption. (Fanny)*

#### Family’s advice and support

When the informants did not gain support from their boyfriend, they would turn to their mother for advice. Three informants followed the advice of their mother and had an abortion. They said:

*Mom advised me definitely not to keep the fetus. She said that I would not be able to raise a baby, and that it is a responsibility for life. (Candy)*

*I followed my mother’s advice and chose to get an abortion. My mom said that I was still too young to look after a baby, so I aborted the fetus. (Ivy)*

Jenny and her boyfriend wanted to keep the baby. However, her parents advised her to get an abortion because she was only 15 and did not want her boyfriend to be prosecuted for having sexual intercourse with a girl under the age of 16. She explained:

*I had no other choice, as I hadn’t reached the age of consent. My parents told me that since I was not of age, if I delivered the baby, my boyfriend would be put in jail. So I agreed to abort the fetus. (Jenny)*

Helen had a poor relationship with her family and had left home at the time of her pregnancy, so she did not discuss it with her family. However, her boyfriend suspected that the baby was not his and forced Helen to undergo an abortion at around five months gestation. Gi Gi, Lucy, and Kitty aborted the fetus soon after getting pregnant, without discussing the matter with anyone. Kitty had only known her boyfriend for a short time, and feared being reprimanded by her parents.

#### Practical considerations

The ability to raise a baby and financial considerations were the major concerns for most of the informants. Two informants, Daisy and GiGi, obtained such childcare support from their boyfriend and financial support from their mother or mother-in-law. They said:

*I believed that my boyfriend was able to earn the money needed to support the baby. His mom could help me to take care of the baby. It was even better that my mom said that she would help me care for the baby. (Daisy)*

*I had concerns about our ability to cope financially, but my boyfriend had a stable income, so I was not worried. My mother-in-law to be would help with child care. (GiGi)*

Lucy could not receive any support from her boyfriend or her own family, but she chose to become a single parent. She moved into a hostel for pregnant girls during her pregnancy and obtained a social security allowance from the government. She said:

*A friend of mine gave me the phone number of a social worker at the girls’ hostel; the social worker then arranged for me to move into the hostel. Then, she helped me to apply for a government allowance. (Lucy)*

Fanny gave birth to the baby, but later separated from her boyfriend. She could not take care of the baby as she was in drug rehabilitation treatment and had no financial support. She was left with no other choice but to give the baby up for adoption. She said:

*I thought about becoming a single parent, but realized that I did not have the ability to do so. I didn’t have the ability to earn money, so I decided to give the baby up for adoption. I wish I had had an abortion. (Fanny)*

Two informants were not ready to be mothers. Neither of them received any support from the putative father or their family members, and so they decided to abort the fetus. Ivy and Kitty said:

*My boyfriend ran away when he found out about my pregnancy. I was still studying and had no job. I was unable to take care of the baby myself. (Ivy)*

*I had only known him for a short time, so we could not stay together just for the baby. I was also too young, and could not manage to care for the baby and to assume the burden of the family finances. No one was there to help. (Kitty)*

#### Personal values in life

Three other informants were against the option of adoption, and chose parenting instead of abortion as they regarded the fetus as a “living being with life”. Daisy and GiGi initially preferred to undergo an abortion, but changed their minds due to the “existence” of the baby. They said that it was not only because they could receive support from their boyfriends but also due to their feelings about the life of the fetus. They explained:

*All of my family and friends suggested that I have an abortion. They were concerned about me, but not about the fetus. I felt “life” inside me; I didn’t want to “kill” it. I thought that since I had made it happen, I had to keep and give birth to the baby. (Daisy)*

*I had thought of abortion in the first month, but as the baby grew gradually in my belly, I didn’t want an abortion any more. When I felt unhappy, the fetus kicked me as if it was comforting me. (GiGi)*

Lucy separated from her boyfriend before she realized that she was pregnant, but still chose to become a parent as she considered it wrong to take the life of a fetus by abortion. She remarked that she respected life and that a fetus has a right to live. She said:

*Previously, I was prejudiced against single mothers; however, I also strongly believed that the fetus has a right to live, and I respect life. I would have felt sorrow if I had given the baby up for adoption. It is also irresponsible. I understood that I had made a mistake, so I told myself that I would be a good mother. (Lucy)*

Even those who chose to get an abortion generally viewed the fetus as having “life”, but they felt that they had no other choice but to scarify the fetus, that they had weighed their other choices and that there were other more important considerations. They explained:

*The baby was innocent and had life. I didn’t really agree with abortion. But if I could not give a good life to my baby, and a father, then it was better that the baby should not suffer. (Candy)*

*I didn’t want an abortion, as the baby was mine and had life. My boyfriend insisted on an abortion, threatening to leave me if I did not get one. Since I didn’t want to lose him, I complied with his request. (Helen)*

*Abortion is like killing. But my boyfriend disappeared; I needed to continue with my life, either study or work, and could not wait to settle the pregnancy before it was too late. (Ivy)*

*I felt bad about the abortion. But I had no other choice as I hadn’t reached the legal age of consent, and my boyfriend would get into trouble, so I had an abortion. (Jenny)*

#### Views on adoption

None of the informants chose to give birth to a baby for adoption. Candy, Ivy, and Kitty held a negative view of adoption. They rejected the idea of giving the baby to others, and worried whether or not their baby would be treated nicely by his/her adoptive parents. They said:

*I wondered how someone could place their baby in an orphanage or put it up for adoption; I would regret not knowing whether the baby was leading a good life, and would keep worrying about whether they were not being treated well. (Candy)*

*The social workers only told me that I could surrender the baby for adoption immediately after delivery, but they wouldn’t tell me anything more about the adoption process and to whom the baby would be given. I just could not see how I could keep thinking that my child was somewhere in this world, but I wouldn’t know how s/he was being treated, and if s/he would blame me for not taking care of him/her. It was better not to give birth than to have to worry about all of these things. (Ivy)*

Daisy, GiGi, and Lucy also held negative views on adoption, in that they believed it would cause psychological distress to the baby. They also believed that they had a responsibility to the baby. They would rather terminate the pregnancy than surrender the baby for adoption. They explained:

*I would rather abort the fetus than give birth to the baby for others to adopt. Adoption would greatly impact the baby’s psychological health, and the baby would grow up to wonder why they had been abandoned by their mother. I am not dead, and I am the baby’s mom: why would I want to abandon my child? If I didn’t want the baby, then I would not give birth to the baby. (Daisy)*

*It is better to terminate the pregnancy than to abandon the baby after bringing it into this world. If I could not take care of my baby, I would prefer to put him/her in an orphanage and visit regularly; there is no need to give the baby up for adoption. (GiGi)*

*I didn’t want my baby to be adopted. I would be sorry and heartbroken; it seemed so irresponsible. (Lucy)*

Fanny was the only one who finally placed her baby for adoption when she separated from her boyfriend. She also had a negative view of adoption. She regretted giving birth to the baby. She explained:

*Adoption meant I would never see my baby again. I would not have delivered the baby had known that I could not keep the baby. Why did I continue the pregnancy? (Fanny)*

## Discussion

Pregnant teenagers took various matters into consideration in making their pregnancy resolution. These interviews revealed that those who decided to end their pregnancy had a less stable relationship with their boyfriends, accepted their family’s advice and support (among those with a “relatively better” relationship with their family), or placed less value on “life”. Those who resolved their pregnancy by deciding to become a parent usually considered that they had a stable relationship with their boyfriend, and had financial and childcare support (practical considerations). Adoption was a less common option for the teenagers.

The relationship with the boyfriend had the greatest impact on a teenage girl’s decision on how to resolve her pregnancy. The boyfriend, the putative father of the baby, was the first person whom all of the informants approached about their pregnancy. Another study has also suggested that adolescent girls seek their romantic partner’s input on an unplanned pregnancy [7]. A stable relationship was the most important consideration of pregnant teens in choosing to become a parent. However, a change in their relationship with their partner would result in a change in their decision on how to resolve the pregnancy. One of the informants, who originally planned to co-parent her child, finally put the baby up for adoption after breaking up with her boyfriend. Those pregnant teens who did not have a stable relationship were asked by their boyfriend to get an abortion.

A study has been reported that young boys usually do not know how to handle the situation when faced with the news of their girlfriend’s pregnancy [2]. This is reflected in this study, where the boyfriends, when hearing the news from their impregnated girlfriends, grumbled, denied, or even left the informants.

Parental views on how to resolve the pregnancy, particularly the views of the pregnant teen’s mother, had a great influence on the final decision taken by these informants. Those who were living with their family were more likely to tell them about their pregnancy and to follow the advice of their family, particularly that of their mother, to have an abortion. During this life-altering moment, adolescents often turn to their own mother for advice, and she is by far their most significant source of support [17]. More than one-third of the pregnant adolescents reported that they turn to their mother in a crisis, and about one-fourth will turn to their other relatives such as their grandmother, aunt, or cousin [18]. The exception is adolescents who live apart from their parents, who tend not to involve their parents in their decision about how to resolve their pregnancy.

The findings of this study show that adolescents who chose to terminate their pregnancy were those who accept their family’s advice and support, and have a “relatively better” relationship with their family. This differs from the observation in studies conducted in Western countries that adolescents who obtained an abortion were more likely to have had a strained relationship with their own parents than those who decided to carry the baby to term [19], and were more likely to describe themselves as independent, rebellious, and one who enjoys being unattached [20].

Three out of four informants who chose the option of parenting came from a single parent family, had a poor relationship with their family, and had already left home. An unhappy home situation caused pregnant teenagers to consciously or subconsciously look for a different kind of life [21]. They sometimes regarded the unplanned pregnancy as a different option offered to them, and decided to keep the baby to have their own family.

In Hong Kong, an unmarried woman with an illegitimate child faces social stigma and discrimination. Unless the young partners decided to get married, it will be difficult for them to carry the baby to term. A study has shown that adolescents choose to leave school to conceal their pregnancy [4]. This is evidenced by the fact that five out of the nine adolescents in this study chose to get abortion immediately after they learned about their pregnancy. In Western countries, unmarried mothers seem to be more accepted by society, and many can return to school if there is someone to take care of their babies [22].

In considering how to resolve the pregnancy, the financial ability and childcare arrangements of the young romantic partners are the major practical concerns. Teenage mothers are struck by the sudden responsibility of having to take care of a child and the extra financial demands [19]. Those informants who choose to get an abortion or to give up their baby for adoption generally expressed their inability to take care of the baby on their own. While informants who chose to co-parent obtained financial and childcare support from the putative father and their family members, the informant who chose to become a single parent received childcare support from her family and social security from the government to cope financially.

The teenagers’ personal views on life influenced their decision on what to do about their pregnancy. Those who chose to become a parent perceived the fetus as a living being and respected its right to live. Although informants who opted for an abortion also viewed the fetus as a life, they put more weight on other matters, such as the influence of the pregnancy on other aspects of their life. They felt that they had no other choice but to go along with the abortion.

Adoption was not a common option for these teenagers. The one who gave up her baby for adoption had only done so reluctantly, after she had been placed in a halfway house for drug treatment. The pregnant adolescents worried about the wellbeing of the baby and felt that they would be heartbroken to think that there was someone in this world living with their “blood”. This is supported by another study in Hong Kong, which found that seven out of ten adolescents who had given their babies for adoption regretted their decision, and frequently missed their baby, felt that they had abandoned the child, and were miserable for having done so [4]. This is different from the situation in the U.S., where teenagers can visit the adoptive family, and receive yearly updates about the child even after adoption [22].

### Implications of the study

The results of this study showed that, in making their decision on how to resolve their pregnancy, pregnant teens approached their boyfriend and mother, and considered some of the practical issues related to childcare. Nowhere did the adolescents report that they had their boyfriend sit down with their family members (particularly their mother) to discuss the decision. The pregnancy is a crisis or change in life about which a dialogue should take place between the significant others of the adolescents. A better understanding of each party’s perspective would allow for better decision making on the resolution of the pregnancy, which would also minimize the possibility of a change in the decision at a later stage of the pregnancy.

An intervention study launched for teenagers and their mothers found that although the mothers of the adolescents were angry and disappointed to learn about the pregnancy at the beginning, having a chance to express and share their feelings helped them to accept the situation, brought them closer to their daughters, and helped them work together to better deal with the situation [17]. Adolescent girls also expressed the view that the intervention helped their mothers to understand that they are also human beings and entitled to make mistakes, to better understand their feelings, and to open their hearts to make a place for them. It is expected that a similar intervention for the young partners could also provide an opportunity for the young partners to closely re-examine their relationship and the depth of their feelings, and to make a decision that they can live more comfortably with.

### Limitations of the study

Due to the sensitivity of the topic and the difficulties experienced in recruiting subjects, only nine informants were interviewed. All of the informants were recruited from one Maternal and Child Health Centre while they were seeking maternity care for a subsequent pregnancy. These women recalled and shared their stories from the previous three years when they were teenagers. The findings may be subject to recall bias. Readers should be cautious in interpreting the decision-making process reported here by young females who are now more mature and may be better at making sense of what they had done.

None of the informants in this study originally chose adoption, and the one who ended up putting her child up for adoption because she had no other choice may not reflect those who chose the option of adoption in the first place. Needless to say, none of those who resolved their pregnancy by abandoning or killing the baby could be identified, thus they were not included as subjects in this study. However, given the sensitivity of the topic and the disapproval of teen pregnancy in Hong Kong, this study has the merit of having obtained the views of those who are willing to share their private stories.

## Conclusions

Teenage pregnancy resolution is a complex issue. A pregnant teenager’s relationships with her romantic partner and family members, and the support she receives from them, and her personal values about life become intertwined when a pregnancy resolution is made. Pregnancy places adolescents in situations where they are called upon to make difficult decisions, and need guidance, support, and approval from parents or other adults. Appropriate and timely counselling and necessary information on the various options and possible consequences for pregnant teenagers and other people involved would be important in assisting them to make an informed choice.

With a better understanding of how teenagers resolve their pregnancy, health professionals and social workers may offer appropriate advice and support to help pregnant adolescents make an informed choice. When necessary, a referral should be made to social workers, non-governmental organizations that provide support to pregnant teens, or other appropriate services.

Health professionals and school personnel who come into contact with teens should include sex and contraceptive education in their practice to help adolescents to understand their responsibilities, but not belittle their need for intimacy and impose their own values on them. Education is important, including discussions on such pregnancy-related issues as outcomes of pregnancy and the consequences of different options for adolescent boys and girls.

Future studies to explore how adolescent boys decide what to about their girlfriend’s pregnancy may also help to shed light on their experiences and interaction. Further studies are needed to investigate in depth the perspectives of pregnant teenagers on the choices of abortion and adoption, and whether there are differences across cultures.
